# Transcriptomics and Plant Hormone Analysis Reveal the Mechanism of Exogenous GA_3_ Promoting Reflowering of *Phalaenopsis* ‘Hatuyuki’

**DOI:** 10.3390/ijms262211069

**Published:** 2025-11-15

**Authors:** Xiaohua Ma, Min Yang, Lei Feng, Qingdi Hu, Yaping Hu, Xule Zhang, Jian Zheng

**Affiliations:** 1 Key Laboratory of Plant Innovation and Utilization, Institute of Subtropical Crops of Zhejiang Province, Wenzhou 325005, China; maxiaohua1120@126.com (X.M.); fengl@zaas.ac.cn (L.F.); qingdihu@163.com (Q.H.); huyp@zaas.ac.cn (Y.H.); zhangxl@zaas.ac.cn (X.Z.); 2 College of Life and Environmental Science, Wenzhou University, Wenzhou 325000, China; yymin0909@163.com

**Keywords:** *Phalaenopsis*, GA_3_, reflowering, hormone, molecular regulation

## Abstract

*Phalaenopsis* orchids are globally significant high-value ornamental flowers due to their strange flower shape, gorgeous color, and long flowering period. The successful implementation of reflowering technology is expected to double the economic value of the *Phalaenopsis* industry. This study selected the cultivated variety *Phalaenopsis* ‘Hatuyuki’ as the material to investigate the effects of exogenous gibberellin A_3_ (GA_3_) application (0, 50, 100, 150, and 200 mg/L) on its reflowering. Growth phenotype analysis indicates that exogenous GA_3_ significantly promotes the occurrence of reflowering in *Phalaenopsis* ‘Hatuyuki’ after the first flowering, specifically manifested in elongated leaves, flower bud differentiation, flower stalk growth, and an earlier onset of flowering. The application of exogenous GA_3_ significantly enhances the accumulation of starch, soluble sugars, and proteins in *Phalaenopsis* ‘Hatuyuki’, while inhibiting the synthesis of free fatty acids. Gibberellins (GA_3_, gibberellin A_1_ (GA_1_), and gibberellin A_8_ (GA_8_)), cytokinins (6-Benzyladenosine (BAPR) and Kinetin (K)), and indole-3-acetic acids (IAAs) (tryptamine (TRA), indole-3-acetic acid (IAA)) are the core endogenous hormones responding to exogenous GA_3_ spraying treatment. Transcriptome analysis identified a total of 3891 differentially expressed genes (DEGs). The KEGG enrichment analysis revealed that the most significantly enriched KEGG pathways included ‘Plant hormone signal transduction’. Key genes involved in the plant hormone signal transduction pathway (*AUX*, *IAA*, *SAUR*, *DELLA*, *MYC2*) were validated through qRT-PCR, suggesting that these genes may be crucial for the exogenous GA_3_ application that promotes the reflowering of *Phalaenopsis* ‘Hatuyuki’. Additionally, this study highlights 202 core DEGs responsive to exogenous GA_3_. Combined with the analysis of hormone signaling pathways, it provides a new perspective for uncovering the key molecular modules involved in GA_3_-regulated reflowering of *Phalaenopsis* ‘Hatuyuki’. Overall, the findings of this study indicate that exogenous GA_3_ application can promote the re-flowering of *Phalaenopsis* ‘Hatuyuki’.

## 1. Introduction

*Phalaenopsis* orchids, belonging to the genus *Phalaenopsis* in the family Orchidaceae, are characterized by their unique floral morphology, prolonged flowering duration, and vibrant coloration-features that have conferred upon them the accolade of the “Queen of Orchids” [1,2]. Within the Orchidaceae family, *Phalaenopsis* is the most widely cultivated and commercially popular genus, supported by its irreplaceable aesthetic value [1]. Due to their exceptional ornamental appeal, *Phalaenopsis* orchids have gained substantial market preference, positioning them as one of the most in-demand and rapidly developing ornamental floral taxa in the global flower market [3]. In recent years, alongside the expansion of the *Phalaenopsis* orchid cut flower market, market demand for cultivars capable of multiple flowering events within a compressed time frame has increased substantially [4]. Nevertheless, in *Phalaenopsis* orchid production, challenges including prolonged nutrient accumulation cycles and low re-flowering rates frequently emerge, which are attributed to inherent genotypic traits and suboptimal cultivation management practices. Consequently, investigating the re-flowering process, associated physiological responses, and underlying molecular mechanisms of *Phalaenopsis* orchids holds significant scientific and practical importance. Among *Phalaenopsis* cultivars, *P*. ’Hatuyuki’ is widely used in the commercial production of *Phalaenopsis* cut flowers due to its characteristics of summer flowering, fresh color, and stable reflowering potential. These traits make it an ideal experimental material for studying the regulatory mechanism of *Phalaenopsis* reflowering, ensuring that the findings of this study have direct implications for industrial application.

Reblooming refers to the phenomenon where plants that typically flower only once a year bloom again outside of their normal flowering period [5]. The occurrence of this phenomenon has been reported in various plants, including *Iris germanica* [6], tree peony [7], *Cercis canadensis* [8], and Rosa [9]. Previous researches indicate that reblooming in plants is significantly influenced by the nutrient content within the plant, photoperiod pathways, gibberellin pathways, and is promoted through the synergistic action of multiple metabolic pathways [5,6,7]. For instance, Wang et al. (2020) demonstrated that defoliation treatments and hormonal fluctuations modulate the transport and metabolism of sugars, thereby exerting a regulatory effect on flower bud development and secondary flowering in peonies [10]. In another study, Li et al. (2024) identified key genes associated with the secondary flowering of *Hemerocallis fulva* via transcriptome analysis; their findings indicated that the GA signaling pathway might serve as one of the core pathways governing the reblooming process of *Hemerocallis* species [11].

Reblooming in *Phalaenopsis* orchids is a specific phenomenon. It occurs in plants that have finished their initial flowering cycle. After removing spent inflorescences, these plants produce new flower spikes. Importantly, this happens outside the typical flowering period. To date, temperature and light are the most extensively studied environmental factors. They influence the flowering of *Phalaenopsis* orchids [12]. Besides, nutrients, plant growth regulators, and cultivation substrates also play significant regulatory roles [2,13,14]. Flowering is a critical developmental transition for plants. It marks the shift from vegetative growth to reproductive growth. For *Phalaenopsis* orchids, this transition is mainly governed by two key regulatory factors. These factors are temperature signaling and hormonal modulation [2]. First, *Phalaenopsis* species need to respond to specific low-temperature stimuli. Only then can they initiate and activate floral bud differentiation programs [2,13]. Notably, temperature-mediated flowering regulation is one of the core techniques. It is used in the commercial production of *Phalaenopsis* orchids. Second, hormonal regulation relies on the exogenous application of plant growth regulators. The purpose is to modulate endogenous hormone homeostasis. This further regulates the expression of genes related to floral bud differentiation. Ultimately, it facilitates flowering [14].

Existing studies have demonstrated that exogenous application of GA_3_ can significantly accelerate the transition of plants from vegetative to reproductive growth, reduce their dependence on low-temperature induction, and improve flower quality [15]. GAs constitute a large family of diterpenoid plant hormones, with over 130 naturally occurring members identified to date; among these, GA_1_, GA_3_, gibberellin A_4_ (GA_4_), and gibberellin A_7_ (GA_7_) are recognized as biologically active forms that predominantly regulate plant growth and development processes, including seed germination, stem elongation, and floral transition [16,17]. In the context of flowering regulation, the GA signaling pathway acts as a core regulatory module across plant species: GAs bind to the receptor *GID1* (GIBBERELLIN INSENSITIVE DWARF1), triggering the degradation of *DELLA* repressor proteins-key negative regulators of flowering. The removal of *DELLA* inhibition activates downstream transcription factors (e.g., PIF, SPL) that modulate the expression of floral meristem identity genes (e.g., FT, SOC1) and floral organ development genes, thereby promoting the transition from vegetative to reproductive growth [15,17,18]. For instance, in Arabidopsis, GA-mediated *DELLA* degradation induces FT expression in leaves, initiating floral induction; in woody plants such as apple, GAs regulate flowering by balancing the expression of floral promoters and repressors [19,20]. Within the Orchidaceae family, exogenous GA_3_ treatment has been verified to effectively induce early flowering and enhance flower quality in *Paphiopedilum callosum* [21].

For *Phalaenopsis* species specifically, low-temperature exposure and plant hormone treatments are generally recognized as viable strategies to regulate floral bud formation and development [22]. However, Low-temperature-based regulatory strategies for plant flowering exhibit a strong reliance on sophisticated environmental control systems, which inherently incur substantial operational cost. Consequently, when applied to large-scale commercial production systems, this approach tends to compress profit margins. In contrast, plant hormone-based treatments offer distinct advantages, including shorter regulatory cycles and higher economic efficiency-key considerations for industrial production. Abundant research has further identified GAs as one of the core hormone classes modulating flowering processes in *Phalaenopsis* [21]. Although exogenous GA_3_ application cannot replace light induction for floral bud initiation in *Phalaenopsis*, it can substitute for the low-temperature requirement necessary to trigger flowering, this enables the elongation of flower stalks and subsequent blooming of *Phalaenopsis* even under high-temperature conditions [19]. Notably, the molecular and physiological mechanisms underlying gibberellin-mediated regulation of reblooming in *Phalaenopsis* remain poorly characterized.

A growing body of research has identified numerous genes involved in regulating hormone-mediated flowering and growth and development processes in plants. Specifically, GAs are known to modulate flowering by orchestrating the spatial expression patterns of core floral regulatory genes, including Twin sister of ft (*TSF*); Squamosa promoter binding protein-like (*SPL*); and Flowering locus t (*FT*) [18,19]. Notably, the biological effects of exogenous GA_3_ or endogenously bioactive GAs exhibit interspecific variation. For instance, in sweet oranges (*Citrus sinensis*), GAs suppress flowering by downregulating the expression of the *Citrus inflorescence meristemless* (*CIFT*) gene in leaf tissues [23]. In saffron (*Crocus sativus*), transcriptomic analyses have revealed that the GA biosynthesis gene *GA3ox1* and *GA2ox1* are significantly upregulated during the floral bud differentiation stage [23]. For orchids, reflowering and flower spike development are jointly governed by genetic regulatory networks and environmental stimuli [23]. However, prior studies on orchids have predominantly focused on the macroscopic phenotypic effects of plant hormones, with limited progress in dissecting the underlying molecular mechanisms [13,14]. Specifically, only a small subset of *Phalaenopsis* flowering-related genes has been validated to be associated with the GA signaling pathway, and the precise molecular mechanism through which exogenous GA regulates reblooming in *Phalaenopsis* remains largely elusive.

Currently, a large body of research leverages multi-omics integrative analysis to dissect molecular regulatory mechanisms, with the aim of elucidating the intrinsic processes that drive floral induction and development in plants. Accordingly, the present study investigates the key regulatory factors governing *Phalaenopsis* reblooming by characterizing changes in floral bud anatomical structure, physiological nutrient profiles, endogenous hormone concentrations, and transcriptomic landscapes under exogenous GA_3_ treatment. This research not only yields novel insights into the GA_3_-mediated regulatory mechanism underlying *Phalaenopsis* reblooming but also constructs a theoretical basis for optimizing the balance between flowering efficiency and economic costs in *Phalaenopsis* cultivation systems.

## 2. Results

### 2.1. Plant Growth

**Figure 1:**
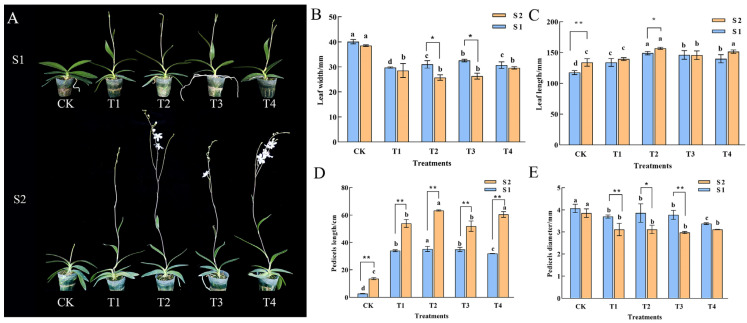
Effects of exogenous GA_3_ on growth of *Phalaenopsis* ‘Hatuyuki’ during reflowering at S1 (60 days post-treatment) and S2 (80 days post-treatment). (**A**) The overall shape of the plant, (**B**) Leaf width (mm), (**C**) Leaf length (mm), (**D**) Pedicel length (cm), and (**E**) Pedicel diameter (mm). Error bars are the standard deviation of the mean (*n* = 3). * indicates significant difference (*p* < 0 .05) between two periods in the same treatment, ** indicates extremely significant difference between two periods in the same treatment, (*p*  <  0.01). Different lower letters indicate statistical differences among different treatment groups at the same period (*p* < 0.05).

Growth morphology of *Phalaenopsis* ‘Hatuyuki’ was observed at the S1 (spike emergence) and S2 (initial flowering) stages following exogenous GA_3_ application (Figure 1). It was found that during the S1 period, the CK plants remained in the vegetative growth stage, while the flower spikes in the GA_3_-treated groups exhibited significant elongation. By the S2 stage, the CK plants were still in the spike emergence stage and had not yet flowered, whereas all GA_3_-treated groups (T1–T4) had successfully entered the initial flowering stage. These results indicate that exogenous GA_3_ application significantly modulates the growth and development of *Phalaenopsis* ‘Hatuyuki’, accelerating the transition from vegetative to reproductive growth. Leaf length, leaf width, flower spike length, and flower spike diameter can intuitively reflect the growth status of the plants. Compared with the CK group, leaf length in GA_3_-treated groups was significantly increased, while leaf width was significantly decreased, suggesting that GA_3_ induces a “narrow and elongated” trend in the leaves of *Phalaenopsis* ‘Hatuyuki’ (Figure 1B,C). The growth of flower spikes was also significantly regulated by GA_3_ (Figure 1D,E). Although the flower spike length in GA_3_-treated groups was significantly greater than that of the CK group, the diameter was significantly smaller than that in CK. This implies that GA_3_ promotes the elongation of flower spikes in *Phalaenopsis*, thereby facilitating its growth and development, but the flower spikes induced by GA_3_ exhibit more slender morphological traits compared to the control group.

### 2.2. Morphological and Cytological Characterization of Spike

**Figure 2:**
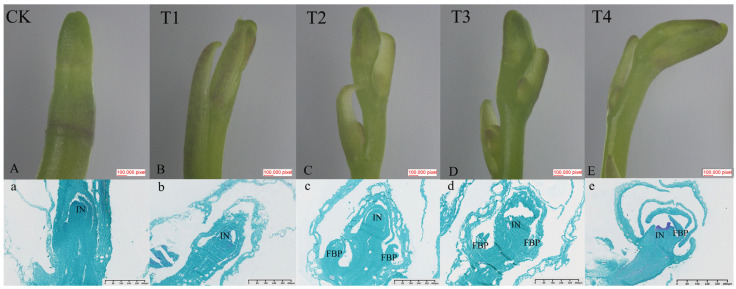
Morphological characterization of spike development in *Phalaenopsis* ‘Hatuyuki’ at S2 period under different GA_3_-treated groups (CK, T1, T2, T3, T4). (**A**–**E**) The morphology of the pedicel under the microscope; (**a**–**e**) Paraffin sections of bud/spike tip tissues under different GA_3_-treated groups. IN: inflorescence primordium, FBP: Flower primordium.

To investigate the effects of exogenous GA_3_ on the growth and development of flower spikes in *Phalaenopsis* ‘Hatuyuki’, paraffin sections of buds and inflorescence tissues at the S2 stage were prepared following exogenous GA_3_ application. Meristematic tissues during inflorescence development were subsequently observed (Figure 2). This study revealed that at the S2 stage, flower buds in the control group (CK) had transitioned from vegetative to reproductive growth, thus entering the inflorescence primordium differentiation phase (IN) (Figure 2A,a). At this stage, the growth cone consisted of two components: the bract primordium and floral primordium. The bract primordium was characterized by a two-layered surface cell structure, with significantly fewer cell layers compared to the initial differentiation stage. Its cells were large and loosely arranged. The outer base of the bract showed a slight outward protrusion, with tightly arranged cells exhibiting active division and deep staining, indicating inflorescence primordium formation. Meanwhile, the GA_3_-treated group (T2) showed *Phalaenopsis* ‘Hatuyuki’ at the flower primordium (FBP) differentiation phase, with protruding cells in the small flower primordia characterized by large, dense nuclei, small cell volumes, and tight arrangement (Figure 2B,b). At this stage, the raceme had begun to form. The flower buds treated with GA_3_ in T3 and T4 reached the peak of differentiation and subsequently underwent gradual differentiation into flower buds. These results indicate that GA_3_ treatment significantly promoted the early differentiation and developmental process of flower buds, exerting a marked effect in accelerating differentiation and reproductive growth.

### 2.3. Physiological Characterization of Phalaenopsis ‘Hatuyuki’ Under GA_3_ Treatment

**Figure 3:**
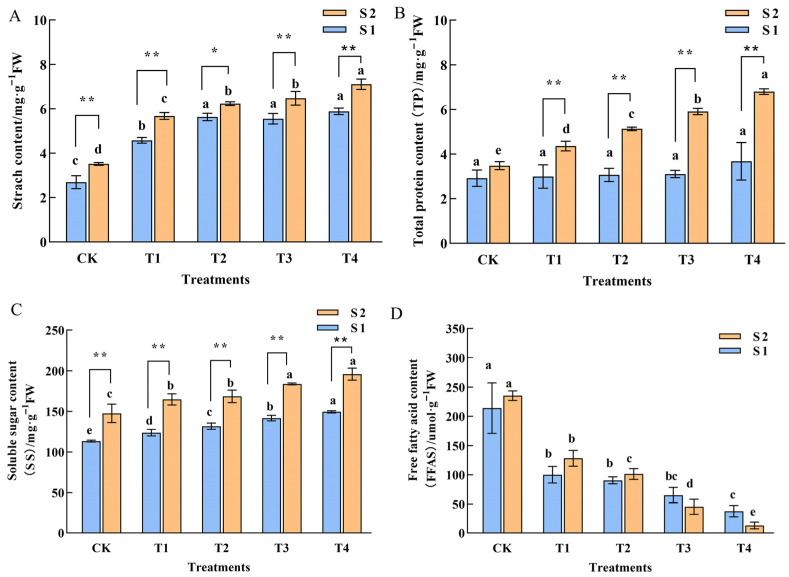
Physiological characterization of *Phalaenopsis* ‘Hatuyuki’ under different GA_3_-treated groups (CK, T1, T2, T3, T4). (**A**) Starch content, (**B**) Total protein content, (**C**) Soluble sugar content, (**D**) free fatty acid content. Error bars are the standard deviation of the mean (*n* = 3). * indicates significant difference (*p* < 0 .05) between two periods in the same treatment, ** indicates extremely significant difference between two periods in the same treatment, (*p* <  0.01). Different lower letters indicate statistical differences among different treatment groups at the same period (*p* < 0.05).

The theory of the carbon-to-nitrogen ratio in plants highlights the crucial role of carbohydrates in the differentiation of floral buds. Starch, formed through the polymerization of the direct products of photosynthesis, serves as a vital substance for the plant’s life activities. This study revealed that following exogenous GA_3_ treatment, the starch content during both the S1 and S2 periods in all treatment groups was significantly higher than that of the control group. Additionally, within the same treatment, the starch content in the S2 period was notably greater than that in the S1 period (Figure 3A). This finding indicates that exogenous GA_3_ significantly enhances the final accumulation of starch in the leaves of *Phalaenopsis* ‘Hatuyuki’. Protein and soluble sugar contents serve as indicators of vigorous plant metabolic activity. This study demonstrated that the total protein content (TP) exhibited dual specificity in its response to GA_3_, varying with both developmental stage and application concentration. During the S1 stage (spike emergence), GA_3_ application induced no significant difference in total protein content of *Phalaenopsis* ‘Hatuyuki’ compared to the control group. However, in the S2 stage, GA_3_ treatment significantly increased leaf total protein content, peaking at the T4 treatment, which was significantly higher than all other treatment groups. Furthermore, soluble sugar (SS) content showed high responsiveness to GA_3_ treatment application, with a significant induction effect observed. In both S1 and S2 stages, SS content exhibited a clear upward trend with increasing GA_3_ concentrations, peaking at the T4 treatment, indicating that GA_3_ application significantly promotes the accumulation of soluble sugars. Additionally, it was observed that the SS content in the S2 period was significantly higher than that in the S1 period, aligning with the material demands of the plant as it transitions into the flowering stage. Free fatty acids (FFAS) play a crucial role in maintaining cell membrane stability and regulating osmotic pressure in plant leaves. This study revealed that during both S1 and S2 stages, FFAS levels in all GA_3_-treated groups (T1–T4) were significantly lower than those in the control group (CK). Notably, FFAS contents across different GA_3_-treated groups exhibited no significant variation between the S1 and S2 stages. These findings suggest that GA_3_ treatment significantly either inhibits FFAS accumulation in leaves or enhances their utilization as energy substrates.

### 2.4. Changes in Endogenous Hormone Contents

**Figure 4:**
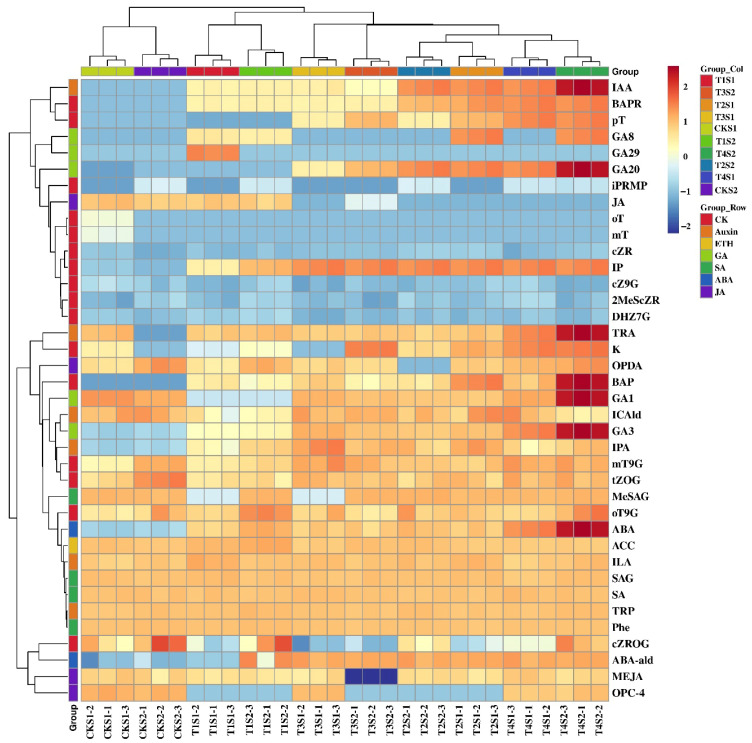
Heatmap of 38 endogenous hormones of *Phalaenopsis* ‘Hatuyuki’ under different GA_3_-treated groups (CK, T1, T2, T3, T4).

The types and levels of endogenous hormones significantly influence various life activities in plants, including growth and development, flower bud differentiation, and flowering. To elucidate the dynamic changes in endogenous hormones in *Phalaenopsis* ‘Hatuyuki’ induced by exogenous GA_3_, we conducted qualitative and quantitative analyses of 109 types of endogenous hormones across seven major categories in the leaves of *Phalaenopsis* ‘Hatuyuki’ (Supplementary Materials). The results revealed that a total of 38 types of endogenous hormones, including abscisic acid (ABA), cytokinins (CKs), GAs, salicylic acid (SA), jasmonic acid (JA), indole-3-acetic acid (IAA), and ethylene (ETH), were detected in the leaves of *Phalaenopsis* ‘Hatuyuki’ across different GA_3_-treated groups (Figure 4). Specifically, we identified 2 types of ABA, 6 types of IAA, 16 types of CKs, 5 types of GAs, 4 types of SA, 4 types of JA, and 1 type of ETH. Additionally, we attempted to measure the contents of melatonin (MLT), trans-cinnamic acid (t-CA), indole-3-carboxylic acid (ICA), methyl indole-3-acetate (MEIAA), N6-benzyladenine-9-glucoside (BAP9G), trans-zeatin riboside (tZR), 5-deoxystrigol (5DS), and strigol (ST) (Supplementary Materials). Some of these substances have been examined in similar studies, but they were not detected in this study.

**Figure 5:**
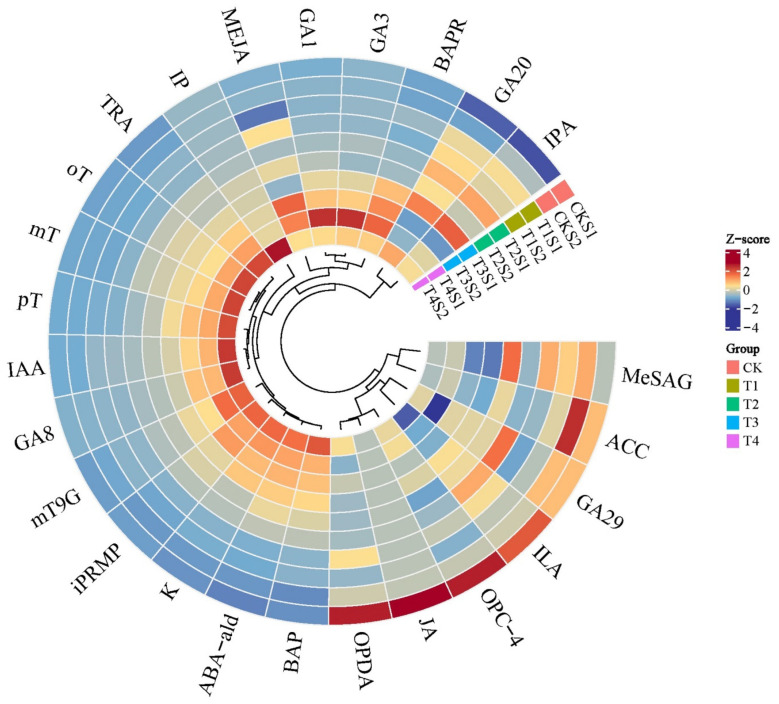
Heatmap of 25 differentially accumulated endogenous hormones of *Phalaenopsis* ‘Hatuyuki’ under different GA3-treated groups at S1 and S2 periods.

Through differential accumulation analysis of all detected hormones, with screening criteria set as |log_2_FC| ≥ 1 and FDR < 0.05, significant differences in endogenous hormone contents were observed. Specifically, a total of 25 hormones with significant differences were identified in the leaves of *Phalaenopsis* ‘Hatuyuki’ under different GA_3_-treated groups within S1 and S2 periods, including five GAs (GA_3_, GA_1_, GA_8_, gibberellin A_20_ (GA_20_), gibberellin A_29_ (GA_29_)), four JAs (methyl jasmonate (MEJA), cis(+)-12-Oxophytodienoic acid (OPDA), 3-oxo-2-(2-(Z)-Pentenyl) cyclopentane-1-butyric acid (OPC-4), jasmonic acid (JA)), four IAAs (3-Indolepropionic acid (IPA), indole-3-lactic acid (ILA), tryptamine (TRA), indole-3-acetic acid (IAA)), one salicylic acid (2-Methoxycarbonylphenyl beta-D-glucopyranoside(MeSAG)), one abscisic acid-related compound (Abscisic aldehyde (ABA-ald)), nine CKs (meta-Topolin-9-glucoside (mT9G), 6-Benzyladenine (BAP), 6-Benzyladenosine (BAPR), Kinetin (K), N6-isopentenyladenine (IP), N-6-iso-pentenyladenosine-5′-monophosphate (iPRMP), para-Topolin (pT), ortho-Topolin (oT), meta-Topolin (mT)), and one ethylene (1-Aminocyclopropanecarboxylic acid (ACC)) (Figure 5).

**Figure 6:**
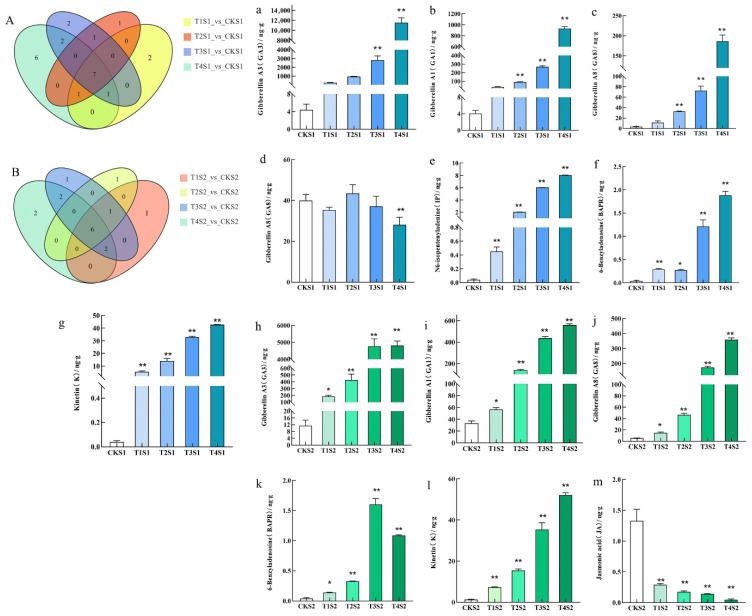
Differential accumulation of endogenous hormone content of *Phalaenopsis* ‘Hatuyuki’ under different GA3-treated groups at S1 and S2 periods. (**A**) S1 period differential accumulation Venn diagram, (**B**) S2 period differential accumulation Venn diagram, (**a**–**g**): The specific hormone types and hormone content of co-differentially accumulated in the S1 period, (**h**–**m**): The specific hormone types and hormone content of co-differentially accumulated in the S2 period. Error bars are the standard deviation of the mean (*n* = 3). * indicates a significant difference from the control (*p* < 0 .05), ** indicates extremely significant difference from the control (*p* <  0.01).

During the S1 period, Venn diagram-based analysis of differentially accumulated endogenous hormones among distinct treatment groups (T1S1 vs. CKS1, T2S1 vs. CKS1, T3S1 vs. CKS1, T4S1 vs. CKS1) identified a total of seven common differential endogenous hormones, namely GA_3_, BAPR, GA_1_, K, IP, GA_8_, and GA_29_ (Figure 6). In the S2 period, an analogous Venn diagram analysis of differentially accumulated endogenous hormones across the treatment groups (T1S2 vs. CKS2, T2S2 vs. CKS2, T3S2 vs. CKS2, T4S2 vs. CKS2) revealed six common differential endogenous hormones: GA_3_, BAPR, GA_1_, K, Jasmonic acid (JA), and GA_8_ (Figure 6). GA_3_, GA_1_, GA_8_, BAPR, and K are differential hormones common to both periods, with GA_3_, GA1, and GA8 belonging to the GAs class, while BAPR and K belong to the CKs class. Specifically, the content of GA_3_, GA1, GA8, BAPR, and K in the leaves of *Phalaenopsis* ‘Hatuyuki’ in GA_3_-treated groups was significantly higher than that in the control group, reaching the highest values during the T4 treatment, except for a slight decrease in BAPR content in the T4 treatment group during the S2 period. Furthermore, during the S2 period, the content of JA in GA_3_-treated groups was significantly lower than that in the control group. Among all detected hormones, GA_3_ content remained high in both periods. These results indicate that the exogenous application of GA_3_ significantly promotes the increase in endogenous GAs and CKs in *Phalaenopsis* ‘Hatuyuki’, preparing for the growth and flowering of the flower spike.

### 2.5. Quality Analysis of Transcriptome Sequencing Data

**Figure 7:**
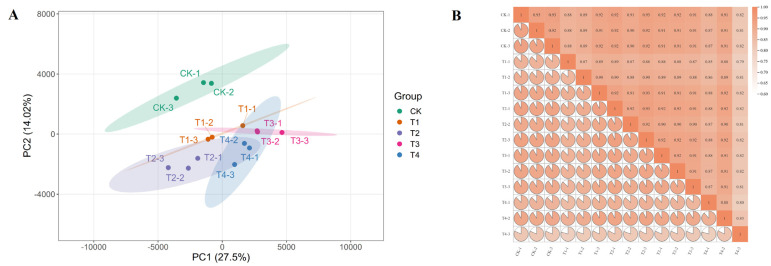
Principal Component Analysis (PCA) and intersample correlation analysis based on gene expression levels. (**A**) Principal Component Analysis (PCA) plot, (**B**) Heatmap of Pearson’s correlation coefficients for gene expression levels among samples.

To elucidate the molecular mechanism by which exogenous GA_3_ application promotes the growth and flowering of *Phalaenopsis* ‘Hatuyuki’, RNA-seq analysis was performed on leaf samples from five treatment groups (each with three biological replicates) at the S2 stage to generate a comprehensive transcriptome profile. Sequencing and assembly metrics are summarized in Table 1. Each sample yielded more than 52 million raw reads, with counts ranging from 52,919,290 to 72,932,666. For all analyzed samples, the Q20 value exceeded 98.99%, while the Q30 value surpassed 96.42%. Across the 15 libraries, the average GC content was 47.184%, with the average proportion of uniquely mapped reads at 9.96%, resulting in the identification of a total of 161,941 Unigenes. By mapping reads to the *Phalaenopsis equestris* genome, approximately 79.22% of known genes were detected across the 15 libraries. Furthermore, principal component analysis (PCA) revealed that samples clustered into five distinct groups corresponding to the five treatment groups (Figure 7A). Pearson correlation analysis indicated strong correlations among the three replicates of each sample (Figure 7B), demonstrating high differentiation among samples and confirming the high quality of the transcriptome data.

### 2.6. Differentially Expressed Genes Screening and Analysis

**Figure 8:**
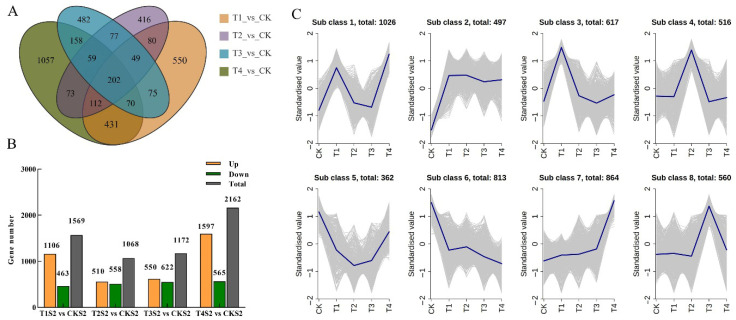
Characterization of differentially expressed genes (DEGs) identified by pairwise comparisons among the different GA_3_-treated groups (CK, T1, T2, T3, T4) in *Phalaenopsis* ‘Hatuyuki’ at S2 period. (**A**) Venn diagram of the DEG numbers in the four pairwise comparisons (T1 vs. CK, T2 vs. CK, T3 vs. CK, and T4 vs. CK). (**B**) Numbers of upregulated and downregulated DEGs in each pairwise comparison. (**C**) Kmeans cluster analysis for all DEGs (class = 8).

To characterize the gene expression response patterns to varying concentrations of exogenous GA_3_ treatment, we performed a comprehensive analysis of differentially expressed genes (DEGs) by comparing each treatment group (T1–T4) with the control group (CK). A total of 3891 DEGs were identified, including 1569 DEGs in T1 vs. CK (106 upregulated and 463 downregulated), 1068 DEGs in T2 vs. CK (558 upregulated and 510 downregulated), 1172 DEGs in T3 vs. CK (622 upregulated and 550 downregulated), and 2162 DEGs in T4 vs. CK (1597 upregulated and 565 downregulated) (Figure 8B). Furthermore, a Venn diagram was utilized to visualize the overlapping DEGs across different comparison groups. The results showed that the four GA_3_ treatment groups collectively responded with 202 core DEGs (Figure 8A), indicating that these DEGs are key regulatory components induced by exogenous GA_3_ application, which may contribute to promoting the growth and development of *Phalaenopsis* ‘Hatuyuki’.

Through K-means clustering analysis, 3891 DEGs were categorized into eight classes (Subclasses 1–8) based on their expression patterns under varying exogenous GA_3_ concentrations (CK, T1–T4) (Figure 8C). The results indicated that the genes in Subclass 2 (*n* = 497) exhibited a significant upregulation in expression during the CK → T1 phase, with normalized values increasing from −1 to approximately 0.5. This subclass maintained a consistently high expression level during the T1 → T2 phase, characterized by minor fluctuations, with only a slight decline observed during the T3 phase. Coupled with phenotypic observations, it was found that GA_3_ treatment during the S2 stage of *Phalaenopsis* ‘Hatuyuki’ under T1–T4 GA_3_-treated concentrations significantly promoted the growth and development of flower spike, with the flower spike length peaking at T2, representing a 364.12% increase compared to CK. This growth pattern was completely synchronized with the expression pattern of Subclass 2 genes, suggesting that the activation of this class of genes may play a crucial role in GA_3_-promoted flower spike growth and development.

### 2.7. GO and KEGG Enrichment Analyses of DEGs

**Figure 9:**
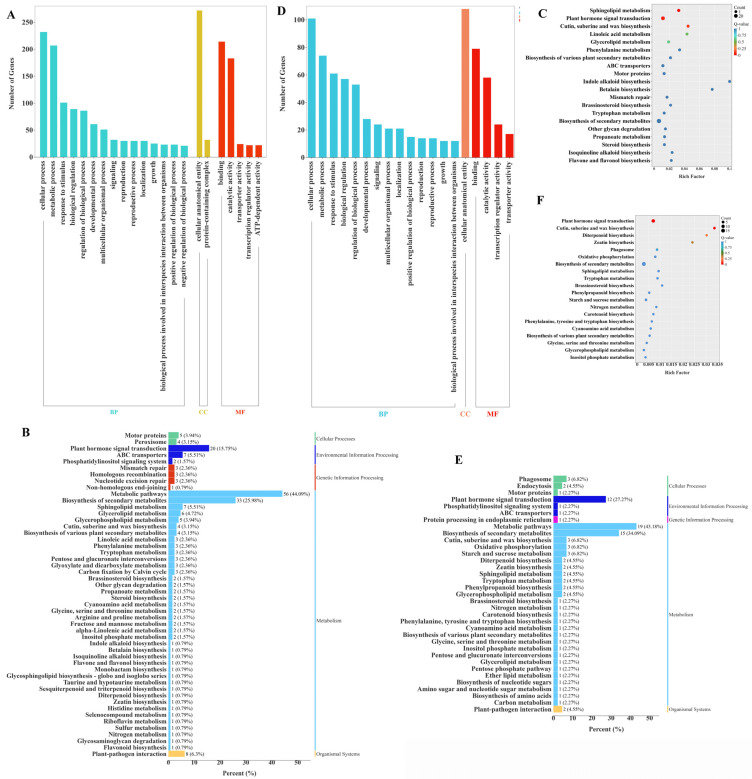
Gene Ontology (GO) enrichment and KEGG pathway annotation. (**A**) Gene Ontology (GO) enrichment of Kmeans clustering analysis (Sub class 2), (**B**) KEGG pathway enrichment of Kmeans clustering analysis (Sub class 2), (**C**) KEGG pathway annotation (Kmeans, Sub class 2), (**D**) Gene Ontology (GO) enrichment of from venn diagram common difference genes, (**E**) KEGG pathway enrichment of venn diagram common difference genes, (**F**) KEGG pathway annotation (venn diagram, 202 difference genes).

To explore the regulatory network of exogenous GA_3_ treatment on the growth and development of *Phalaenopsis* flower spikes, Gene Ontology (GO) and Kyoto Encyclopedia of Genes and Genomes (KEGG) functional enrichment analyses were performed on 497 genes from Kmeans clustering analysis (Sub class 2) and 202 DEGs that commonly responded to GA_3_ treatment (Figure 9). The results indicated that among the 497 genes from Kmeans clustering analysis (Sub class 2), the most enriched GO terms were ‘cellular process’ and ‘metabolic process’ in the biological process category; ‘cellular anatomical entity’ in the cellular component category; and ‘catalytic activity’ and ‘binding’ in the molecular function category. For KEGG enrichment analysis, these DEGs were categorized into five KEGG categories: Cellular Processes, Environmental Information Processing, Genetic Information Processing, Metabolism, and Organismal Systems. “Metabolism” is the most significantly enriched pathway (*p* < 0.05), including “Metabolic pathways” and “Biosynthesis of secondary metabolites” (Figure 9B). The most significantly enriched KEGG pathways include “Plant hormone signal transduction”, “Sphingolipid metabolism,” and “Cutin, suberine and wax biosynthesis”, indicating that these DEGs are involved in the process of exogenous GA_3_ application inducing the development of flower spikes in *Phalaenopsis* ‘Hatuyuki’. The 202 differentially expressed genes (DEGs) that responded collectively in the GA_3_-treated groups (T1, T2, T3, T4) showed similar GO enrichment and KEGG enrichment analysis results as mentioned above (Figure 9D–F). The most enriched GO terms were “cellular process”, “metabolic process”, “response to stimulus”, “biological regulation”, and “regulation of biological process” in the biological process category; “cellular anatomical entity” in the cellular component category; and “catalytic activity” and “binding” in the molecular function category. The most significantly enriched KEGG pathways included “Plant hormone signal transduction” and “Biosynthesis of secondary metabolites”, indicating that these DEGs are core response genes involved in the development of *Phalaenopsis* flower spike induced by exogenous GA_3_ application. Compared with the general studies on GA_3_-regulated plant flowering, the 202 core DEGs identified in this study specifically respond to the reflowering process of *Phalaenopsis*. Their enrichment pattern in the hormone signal transduction pathway provides key targets for clarifying the unique reflowering molecular mechanism of Orchidaceae plants.

### 2.8. Combined Analysis of Endogenous Hormones and Transcriptome

**Figure 10:**
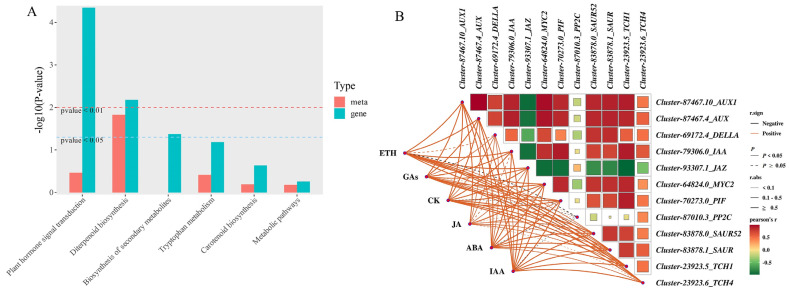
Transcriptome and endogenous hormone combined analysis. (**A**) KEGG co-enrichment pathway analysis, (**B**) Correlation analysis between differentially expressed hormones and plant hormone signal transduction pathway genes.

This study revealed via KEGG pathway enrichment analysis that during the growth and development of *Phalaenopsis* ‘Hatuyuki’ flower spikes regulated by exogenous GA_3_, DEGs and differentially accumulated endogenous hormones (DAMs) were primarily enriched in the plant hormone signal transduction pathway, thereby activating biological functions associated with plant growth, development, and flowering(Figure 10A). To further elucidate the regulatory relationships between differentially accumulated endogenous hormones and genes within the major enriched pathways during the growth and development of *Phalaenopsis* ‘Hatuyuki’ flower spikes, Pearson correlation analysis was performed on six classes of endogenous hormones (ETH, GAs, CK, JA, ABA, IAA) and genes involved in the plant hormone signal transduction pathway, with subsequent filtering of significant correlations and visualization of results(Figure 10B). The associations were depicted using lines-wherein distinct colors and line styles (solid/dashed) denote positive/negative correlations and significance levels-and color blocks representing the magnitude of correlation coefficients. The results showed that different hormones exhibited diverse correlation strengths and significances with genes; endogenous hormones such as GAs, CK, and IAA showed significant positive correlations with genes including *Cluster-87467.10_AUX1*, *Cluster-87467.4_AUX*, and *Cluster-83878.0_SAUR52*. JA exhibited a significant negative correlation with genes such as *Cluster-93307.1_JAZ*. IAA and GAs, except for *Cluster-87010.3_PP2C*, showed statistically significant correlations with other genes.

### 2.9. DEGs Related to Endogenous Hormone Signal Transduction

**Figure 11:**
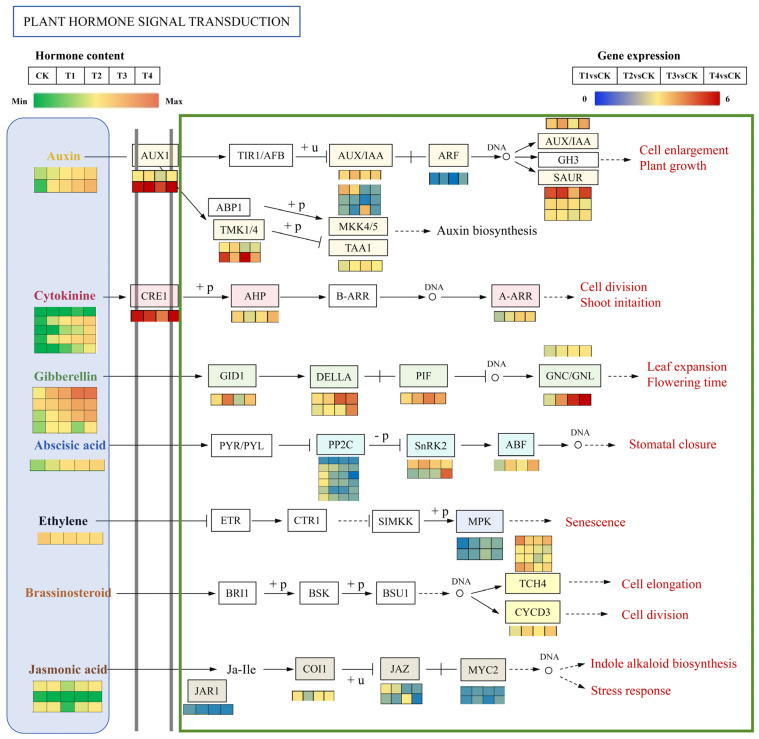
Heatmaps of differentially expressed genes (DEGs) related to plant hormone signaling transduction pathway of *Phalaenopsis* ‘Hatuyuki’ under different GA_3_-treated groups at S2 period. From top to bottom, the seven panels show DEGs involved in the auxins’s, cytokinins’s, gibberellic acids’s, abscisic acids’s, ethylene’s, brassinosteroids’s; and jasmonic acid’s signal transduction pathways, respectively. Orange squares indicate upregulation, whereas green squares indicate downregulation. The color scale corresponds to the average log10 (FPKM + 0.1) and Z-score (normalized by the R software 3.4.3) values.

Building upon the enriched plant hormone signaling pathways, a regulatory network integrating transcriptomic data and endogenous hormone levels was constructed to unravel the molecular mechanism through which exogenous GA_3_ administration facilitates the growth and development of *Phalaenopsis* ‘Hatuyuki’ flower spikes (Figure 11). Figure 11 depicts a heatmap of plant hormone levels in conjunction with heatmaps illustrating signaling pathways and the expression profiles of related genes, elucidating the signaling cascades corresponding to seven hormone classes: IAAs, CKs, GAs, ABAs, ETHs, Brassinosteroid (BR), and JAs. This figure further emphasizes variations in hormone concentrations and the expression patterns of key pathway-related genes across different exogenous GA_3_-treated groups (CK, T1, T2, T3, T4).

In the course of *Phalaenopsis* ‘Hatuyuki’ responding to GA_3_ spraying, two primary differential auxins (TRA and IAA) were identified. With the elevation of exogenous GA_3_ concentration, the levels of TRA and IAA exhibited a significant increase. Within the IAA signaling pathway, eight differentially expressed genes involved in auxin response or transport were detected, encompassing *auxin receptor genes/IAA* (*Aux/IAA*), *auxin response factors* (*ARFs*), and three *auxin-responsive proteins* (*SAURs*). Among these, *Aux1*, *IAA*, and the three *SAURs* were significantly upregulated in the GA_3_-treated groups. Additionally, the auxin signaling branch pathway *ABP1-TMK1/4* regulates *MKK*, which participates in auxin biosynthesis; *MKK* showed significant downregulation in the GA_3_-treated groups. Five cytokinin species (BAPR, K, IP, BAP, pT) displayed differential responses, predominantly exhibiting a significant increase with the escalation of exogenous GA_3_ concentration. In the CK signaling pathway, *cytokinin receptors (CRE)*, response regulatory factors (*ARRs*), and genes encoding aminoglycoside phosphotransferases (*APH*) were significantly upregulated in the GA_3_-treated groups. Moreover, four GAs species (GA_3_, GA_1_, GA_8_, GA_20_) were found to be significantly upregulated when *Phalaenopsis* ‘Hatuyuki’ responded to exogenous GA_3_ spraying.In the GA signal transduction pathway, the GA receptor gene *GID1*, the core response protein *DELLA*, and the *phytochrome-interacting factor 4* (*PIF*) are all upregulated at GA_3_-treated groups. The content of ABA-ald shows significant changes with the increasing concentration of exogenous GA_3_ applied to *Phalaenopsis* ‘Hatuyuki’, increasing at GA_3_-treated groups. The protein kinase *SnRK* involved in ABA signal transduction and the ABA response element binding factor (*ABF*) both exhibit significant upregulation. Ethylene content significantly decreases with the increasing concentration of exogenous GA_3_. Three JAs (OPC-4, JA, OPDA) show significant increases during T3 and T4 treatments. In the JA signal transduction pathway, the *JA receptor JAR1* and *MYC2* are significantly upregulated at GA_3_-treated groups. Furthermore, in the BR signaling pathway, xyloglucan:xyloglucosyl transferase (*TCH4*) and cyclin D3 (*CYCD3*) are significantly upregulated at GA_3_-treated groups, promoting downstream responses in cell division and ultimately contributing to the growth and development of *Phalaenopsis* ‘Hatuyuki’ flower spikes.

### 2.10. qRT-PCR Verification

**Figure 12:**
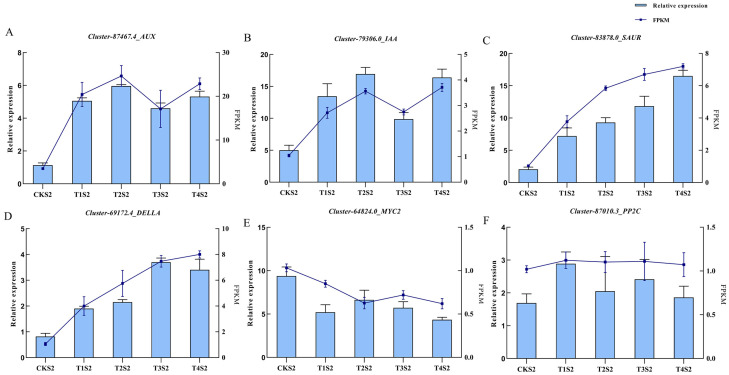
qRT-PCR validation of the expression patterns of 6 differentially expressed genes (DEGs) of *Phalaenopsis* ‘Hatuyuki’ under different GA_3_-treated groups at S2 period. The left y-axis represents the relative expression levels normalized from the qRT-PCR results; the right y-axis represents the FPKM values from RNA-seq. Error bars are the standard deviation (SD) of the mean (*n* = 3). (**A**) *Cluster-87467.4 (AUX)*, (**B**) *Cluster-79306.0 (IAA)*, (**C**) *Cluster-83878.0 (SAUR)*, (**D**) *Cluster-69172.4 (DELLA)*, (**E**) *Cluster-64824.0(MYC2)*, (**F**) *Cluster-87010.3 (PP2C)*.

Based on the primary enriched pathways under varying concentrations of exogenous GA_3_ treatments, six differentially expressed genes were selected for quantitative real-time PCR (qRT-PCR) validation (Figure 12). Among these six genes, three are key components of the IAA signaling pathway (*AUX*, *IAA*, *SAUR*), one is a core gene in the GA signaling pathway (*DELLA*), another belongs to the jasmonic acid signaling pathway (*MYC2*), and the remaining one is a key gene in the ABA signaling pathway (*PP2C*).The results demonstrated a high concordance between qRT-PCR data and transcriptome sequencing results. Specifically, compared with the control group, *AUX*, *DELLA*, *IAA*, and *SAUR* were significantly upregulated in GA_3_-treated groups. *PP2C* expression exhibited no significant differences across treatment groups, while *MYC2* was significantly downregulated in GA_3_-treated groups.These findings suggest that exogenous GA_3_ application triggers perturbations in endogenous hormone homeostasis, thereby activating hormone signaling cascades and inducing the upregulation of a series of pathway-related genes. This molecular cascade subsequently promotes cell division, shoot development, facilitates elongation growth of orchid flower spikes, and ultimately accelerates flowering onset.

## 3. Discussion

*Phalaenopsis* orchids are among the most popular and top-selling ornamental plants in the world. The differentiation of flower buds and the elongation of flower stalks are key steps in the regulation of flowering time in *Phalaenopsis*. Multi-flowering *Phalaenopsis* varieties hold substantial application potential in the cut flower market, as they reduce energy consumption during flowering regulation and enhance economic efficiency [2]. Environmental factors, genetic background, and endogenous hormones exert significant regulatory effects on the re-flowering of *Phalaenopsis* [13,24]. In this study, the small-flowered, pale pink *Phalaenopsis* ‘Hatuyuki’ was selected as the research material. Through the application of exogenous GA_3_ at varying concentrations, coupled with integrated analyses of growth phenotypes, transcriptomes, and endogenous hormone levels, we revealed that the reblooming of *Phalaenopsis* ‘Hatuyuki’ is not regulated by a single factor (e.g., hormone content) but by multiple hormones and their associated genes. Notably, exogenous application of GA_3_ can promote flower bud differentiation, flower spike growth, and flowering advancement.

### 3.1. The Effects of GA_3_ Treatment on the Growth and Physiology of Phalaenopsis ‘Hatuyuki’

GA_3_ is a ubiquitous plant growth regulator, with primary functions encompassing the promotion of flowering, enhancement of fruit set, and inhibition of fruit abscission [16,25]. It also exhibits the capacity to break dormancy in plant organs including seeds and bulbs [26]. In the present study, GA_3_ treatment induced leaf elongation in *Phalaenopsis* ‘Hatuyuki’, while promoting flower spike elongation, accelerating flower spike growth, and advancing flowering time. However, it led to a decrease in flower spike diameter. This phenomenon may be attributed to exogenous GA_3_ inducing rapid growth of *Phalaenopsis* ‘Hatuyuki’ flower spikes, which accelerates secondary blooming and reduces the time window for nutrient accumulation in plants. Consequently, nutrient deficiency occurs, leading to flower spike thinning. Observations from paraffin sections visually demonstrate the growth status of *Phalaenopsis* ‘Hatsuyuki’ flower spikes: GA_3_-treated plants exhibited accelerated vegetative growth, entering the phases of floral bud differentiation and spike emergence earlier. This further confirms that exogenous GA_3_ application can promote reproductive growth in *Phalaenopsis* ‘Hatsuyuki’, thereby facilitating secondary blooming. The findings of Li et al. (2022) are consistent with those of the present study, revealing that GA_3_ can promote flower spike elongation by regulating plant hormone levels, thereby enhancing the flowering quality of *Phalaenopsis* ‘Hatsuyuki’ [24]. However, other studies have demonstrated that GA_3_ application can reduce leaf abscission in *Phalaenopsis* cultivar ‘Amethyst’ [13]. This discrepancy may arise from genetic background differences among *Phalaenopsis* varieties, leading to divergent responses to GAs, or from variations in GAs tolerance across different varieties [27,28].

The advancement of *Phalaenopsis* re-flowering is jointly regulated by multiple physiological factors, with carbohydrates, and free fatty acids playing distinct yet synergistic roles [29]. The findings of this study reveal that GA_3_ significantly enhances the physiological metabolic activity of *Phalaenopsis* ‘Hatsuyuki’, particularly by increasing starch content, soluble sugar (SS) levels, and protein content, while markedly inhibiting the accumulation of free fatty acids in leaves. Numerous studies have established that starch content serves as a critical indicator of a plant’s carbohydrate storage capacity, and the carbon-nitrogen ratio theory pertaining to flower bud differentiation underscores the essential role of carbohydrates [30,31]. Our results showed that leaf starch content in the T4 group decreased by 56.3% at the S1 stage (spike emergence), while soluble sugar content increased by 42.1% (Figure 3). This carbohydrate reallocation-from starch storage to soluble sugar transport—provides sufficient carbon sources for flower spike elongation (spike length increased by 364.12%, Figure 1D) and floral organ development [10,32]. FFAs, which include various types such as linolenic acid, are precursors of JA [33]. A decrease in the total amount of FFAs was detected in the T1–T4 treatment groups; it is hypothesized that this reduction in FFAs may be consistent with a decrease in JA content, and FFAs may function as flowering inhibitors [12,13,24]. GA_3_ may regulate FFAs metabolism to reduce JA biosynthesis, thereby indirectly eliminating the JA-mediated inhibition of *Flowering Locus (FT)* gene expression and ultimately promoting early flowering [8,13,26,28]. These physiological changes collectively explain why the 200 mg/L GA_3_ treatment promoted *Phalaenopsis* re-flowering, highlighting the need to consider multiple physiological factors synergies in optimizing flowering regulation protocols [34,35,36].

From the perspective of commercial *Phalaenopsis* cultivation, the optimized GA_3_ treatment protocol in this study has practical application potential. According to the table and physiological results, for commercially mature plants (with 5–6 leaves, the main seedling age in production), the application of exogenous GA_3_ at a concentration of 150–200 mg/L can advance reflowering by 20–25 days, which is consistent with the results of the S2 stage. This addresses the core demand for supply during the peak period of the cut flower market. Notably, compared with traditional low-temperature induction, this protocol has a significant cost advantage, greatly improving the economic benefits of *Phalaenopsis* cultivation.

### 3.2. The Effect of GA_3_ Treatment on the Content of Endogenous Hormones in Phalaenopsis ‘Hatuyuki’

Endogenous hormone levels in plant leaves are closely associated with flower bud differentiation and growth and development [37]. Accordingly, exogenous GA_3_ treatment can promote flower bud differentiation and growth in *Phalaenopsis* by modulating dynamic changes in endogenous hormones within the plant. Extensive previous research has demonstrated that plant hormones play a pivotal role in regulating flower bud formation in *Phalaenopsis* [13,24]. In this study, 38 distinct endogenous hormones encompassing seven classes were detected in the leaves of *Phalaenopsis* ‘Hatuyuki’, including ABA, CKs, GAs, SAs, JAs, IAAs, and ETHs. This finding indicates that the growth and development of *Phalaenopsis* ‘Hatuyuki’ result from the coordinated regulatory effects of these hormones. Consistent results were reported in Gao ‘s research on fragrant *Phalaenopsis* cultivar ‘Purple Crystal’, ‘Pink Crystal’, and ‘Colorful Doll’, which revealed that hormones such as IAAs, SA, ETH, GAs, ABA, JA, and CKs exert coordinated regulatory control over flower bud differentiation and flower spike growth and development in *Phalaenopsis* [38].

This study further demonstrates that exogenous GA_3_ application significantly upregulates the levels of endogenous GAs (GA_3_, GA_1_, GA_8_, GA_20_, GA_29_), IAAs (IPA, ILA, TRA, IAA), and CKs (mT9G, BAP, BAPR, K, IP, iPRMP, pT, oT, mT) in the leaves of *Phalaenopsis* ‘Hatuyuki’. This suggests that exogenous GA_3_ primarily induces a significant elevation in endogenous GA_3_ levels, which subsequently synergistically upregulates the levels of endogenous IAAs and CKs. The increased auxin and cytokinin levels promote cellular division in orchids, ultimately facilitating flower bud differentiation, flower spike elongation, and flowering advancement. Consistent findings have been documented in previous studies, for example, Pérez-Rojas et al. found that high concentrations of cytokinins (CTK) can induce flower bud formation in strawberry plants [8], while Huang et al. demonstrated that high concentrations of auxin (IAA) promote flower bud formation in *Alfalfa* [39]. However, some studies have demonstrated that high concentrations of GA inhibit flowering progression and bud initiation in *Phalaenopsis* [13], which is inconsistent with the findings of the present study. This discrepancy may stem from marked differences in the genetic backgrounds of distinct *Phalaenopsis* varieties. The current study revealed that the variation trend of endogenous JA induced by exogenous GA_3_ application differed from that of IAAs, presenting a decreasing pattern, whereas ABA showed an increasing trend. Previous studies have indicated that low concentrations of ABA and JA are associated with flower bud activation and flower spike development [13,24].

The growth and flowering period of *Phalaenopsis* are regulated by multiple physiological factors, among which plant hormones play a key regulatory role in physiological processes, ultimately contributing to the advancement of its flowering period [22,24,35]. In this study, exogenous GA_3_ treatment significantly increased the contents of endogenous IAAs and ABA; this hormone balance, characterized by the upregulation of auxin and abscisic acid, accelerated the transition of floral meristems. Meanwhile, the CKs increased significantly under GA_3_ treatment group, which promoted cell division in *Phalaenopsis* flower buds and shortened the flower bud differentiation period. This is consistent with previous findings that a “high ABA and CKs” ratio is a key trigger for the advancement of *Phalaenopsis* flowering period [13,38]. In addition, JAs usually acts as a flowering inhibitor in plants; in this study, the GA_3_ treatment group showed a significant decrease in JA content [13,38]. This decrease may be achieved by inhibiting JA biosynthesis, which indirectly eliminates JA-mediated inhibition of *Phalaenopsis* flowering and ultimately promotes its reflowering [24,25,39].

Further analysis of hormone-gene interaction networks reveals specific crosstalk mechanisms between GAs, IAA, CKs, and JA in regulating *Phalaenopsis* ‘Hatuyuki’ reflowering. Pearson correlation analysis (Figure 10B) shows that endogenous GA_3_ content exhibits a significant positive correlation with IAA and CKs, while displaying a negative correlation with JA. This suggests that GAs not only directly promote IAA and CK accumulation but also inhibit JA biosynthesis, forming a “promotion-inhibition” regulatory loop.

### 3.3. The Plant Hormone Signaling Pathway Regulates the Flower Bud Differentiation and Flower Spike Growth of Phalaenopsis ‘Hatuyuki’

Flower bud differentiation and flower spike development in *Phalaenopsis* orchids involve a series of intricate physiological, biochemical, and molecular biological processes [13]. In recent years, an increasing number of studies have employed transcriptome sequencing technology to investigate the regulatory mechanisms underlying plant flower bud differentiation, revealing that this process is primarily mediated by responses to plant hormones and growth regulators [8,25,36]. In the present study, transcriptome profiles of *Phalaenopsis* ‘Hatuyuki’ following treatment with varying concentrations of GA_3_ were analyzed, leading to the identification of 3891 DEGs. Subsequent K-means clustering analysis of these DEGs identified 497 subclass-specific differentially expressed genes that correspond to the phenotypic growth and developmental trajectories of *Phalaenopsis* ‘Hatuyuki’. Furthermore, KEGG pathway and GO enrichment analyses of these 497 genes revealed significant enrichment of genes involved in plant hormone signaling pathways. Notably, the coordinated expression pattern between the 202 core DEGs identified in this study and hormone signaling pathway genes may represent a unique molecular signature for GA_3_-promoted *Phalaenopsis* reflowering, reflecting the specific regulatory mechanism underlying the reflowering process in Orchidaceae plants. Previous transcriptome studies on *Phalaenopsis* either focused on changes in endogenous hormones, as well as the differential expression of hormone biosynthesis and signaling pathway genes, during the development of *Phalaenopsis* flower stalks, rather than the differential response of hormone signaling pathway-related genes and the analysis of the regulatory network for reflowering mechanism during *Phalaenopsis* reflowering induced by exogenous gibberellin spraying, which is the focus of this study [24,35]. Or they focused on 6-BA-regulated flower bud formation and development, with an emphasis on the roles of CKs and salicylic acid (SA); these studies did not involve the reflowering process and only focused on the physiological and molecular mechanisms of the first flower bud differentiation [13,39].

These findings suggest that exogenous GA_3_ application induces dynamic changes in endogenous hormones in *Phalaenopsis* ‘Hatuyuki’, thereby activating a cascade of transcriptional responses in genes associated with endogenous hormone signaling transduction pathways. This regulatory cascade ultimately promotes flower bud differentiation, flower spike elongation, and reflowering in the orchid. Exogenous GA_3_ application not only elevates endogenous GA levels but also triggers a cascade of regulatory events that modulate IAA accumulation. The accumulated IAA then activates the expression of auxin-responsive genes such as *AUX1* and *SAUR52*, as validated by qRT-PCR (Figure 12). This regulatory module involves GA_3_, which leads to PIN upregulation [11,24]. This upregulation then results in IAA accumulation, and such accumulation causes auxin-responsive gene activation. This entire process promotes cell expansion in flower stalks, and it is consistent with the observed 364.12% increase in flower spike length in the T2 group (Figure 1D). Notably, a similar GA-IAA crosstalk was reported in *Hemerocallis fulva,* where GA_3_ treatment increased IAA content by 42% in flower buds and upregulated *PIN* gene expression, highlighting a conserved role of GAs in modulating IAA transport for flowering regulation [11].

The synergistic increase in CK levels following GA_3_ treatment is attributed to GAs’ dual regulation of CK biosynthesis and degradation, ensuring the accumulation of active CKs to support flower bud differentiation [40,41]. This is supported by a recent study on *Paphiopedilum callosum*, where GA_3_application increased IPT activity by 35% and elevated endogenous CK content by 58%, promoting flower stalk elongation [35]. In our study, BAPR and K content in the T4 group was higher than that in CK (Figure 6), while K content increased approximately by 1.8-fold. The accumulated CKs then bind to cytokinin receptors *(CRE1*) in the flower bud meristem, activating the two-component signaling pathway. This activation induces the expression of type-A response regulators (*A-ARR3*), which synergizes with IAA to promote meristem maintenance and floral organ differentiation. A similar regulatory pattern was reported in strawberry, where GA_3_treatment reduced CKX activity by 28% and increased CK content, inducing flower bud formation [8].

In the GA signaling pathway, the upregulation of genes such as *GID* and *DELLA* ultimately contributes to leaf growth and flowering advancement [39]. In other plant species, such as *Arabidopsis* and rice, reduced GA levels result in increased branching/tillering [17,42,43]; in tea plants, exogenous GA_3_ treatment increases the branching angle of new shoots [44]. These findings indicate that GA effects display interspecific variability. The GA signaling core gene *DELLA* (Cluster-69172.4_DELLA) acts as a hub for this crosstalk. Exogenous GA_3_-induced *DELLA* upregulation thus simultaneously activates IAA/CK signaling (by relieving repression on *ARF* and *A-ARR*) and inhibits JA signaling (by reducing *MYC2* expression) [42,43]. This coordinated regulation ensures that cell elongation (driven by IAA) and cell division (driven by CKs) are synchronized during flower spike growth, while JA-mediated flowering repression is alleviated—collectively accelerating reflowering.

The ABA signaling pathway is likewise implicated in flower bud differentiation and flower spike development in *Phalaenopsis*, evidenced by the high expression of the *SnRK2* gene. This suggests that elevated ABA levels may facilitate flower bud differentiation, a phenomenon also observed in *Pyrus pyrifolia* [45], *Malus domestica* [20], and other plants. JA releases the transcription factor *MYC2* through inducing *JAZ* degradation, and *MYC2* can repress the expression of the *flowering gene* (*FT*) [46]. Consistent with this mechanism, the observed reduction in JA levels and downregulated MYC2 expression in the present study provide empirical support. Notably, while strigolactone was not detected in the samples analyzed in this study, the signaling pathway genes *TCH4* and *CYCD3* were highly upregulated, thereby promoting cell division and contributing to flower bud differentiation and flowering in *Phalaenopsis* ‘Hatuyuki’.

The coordinated regulation of flowering in *Phalaenopsis* ‘Hatuyuki’ by GAs, IAA, CKs, and their associated genes forms a multi-layered hormonal network, with each component playing a specific role in driving floral transition and development. GA_3_-induced *DELLA* upregulation activates ARF, which enhances IAA signaling by upregulating *SAUR* and *AUX* genes. The upregulated IAA further reinforces ARF expression, ensuring sustained cell expansion during flower stalk growth. *DELLA* upregulation also upregulates *A-ARR*, a key gene in CK signaling. Activated *A-ARR* enhances CK-mediated cell division in the apical meristem, which is critical for flower bud differentiation. GA_3_ treatment downregulates JA content (42% lower in T4 vs. CK, Figure 6) and the JA signaling gene *MYC2*. Reduced *MYC2* relieves its repression of FT, allowing FT to be upregulated by GA-activated PIF [40,41]. This synergistic effect of GAs and JA further accelerates floral induction, as FT is a core integrator of flowering signals in plants [19,44].

Quantitative real-time PCR (qRT-PCR) validation confirmed the expression patterns of these key genes: *AUX*, *IAA*, *SAUR*, and *DELLA* were significantly upregulated in GA_3_-treated groups, while MYC2 was downregulated (Figure 12), consistent with transcriptome data. This multi-pathway coordination ensures that Phalaenopsis ‘Hatuyuki’ efficiently transitions to reflowering, with flower bud differentiation and flower stalk growth occurring synchronously [13,24].

## 4. Materials and Methods

### 4.1. Plant Materials and Growth Conditions

The commercially cultivated small-flowered cultivar *Phalaenopsis* ‘Hatuyuki’ was selected as the experimental material, with uniform growth performance (5–6 mature leaves per plant) and completion of the natural flowering period. Plants were grown in a glass greenhouse at the Xiongxi Base of Zhejiang Institute of Subtropical Crops (E120.5497, N27.9683). Greenhouse environmental conditions were controlled as follows: daytime temperature 30 ± 2 °C, nighttime temperature 26 ± 2 °C, relative humidity 50–60%, and natural photoperiod ~12 h.

On 1 August 2024, all *Phalaenopsis* ‘Hatuyuki’ plants post-natural flowering were subjected to old spike removal. Starting from 5 August, exogenous GA_3_ was applied via foliar spraying, with five concentration gradients set: 0 mg/L (control, CK), 50 mg/L (T1), 100 mg/L (T2), 150 mg/L (T3), and 200 mg/L (T4). GA_3_ solution was uniformly sprayed onto leaf surfaces until runoff; applications were performed every 7 days for a total of 7 times. Each treatment included 3 biological replicates (10 plants per replicate), totaling 150 plants. Samples were collected for analysis at two key stages post-treatment initiation: the 60th day (S1 stage, defined as inflorescence emergence) and the 80th day (S2 stage, defined as initial flowering). The samples were immediately placed in liquid nitrogen and stored at −80 °C for subsequent transcriptome analysis and determination of endogenous hormone content.

### 4.2. Growth Measurement

At the S1 (spike emergence) and S2 (initial flowering) stages post-GA_3_ spraying treatment, phenotypic traits of plants across different treatment groups were measured using a standard ruler (for linear dimensions) or vernier caliper (for diameter). The measured traits and their specific measurement criteria were defined as follows:

Leaf length: The maximum straight-line distance from the leaf base to the leaf apex;

Leaf width: The width at the widest region of the middle leaf segment;

Flower spike length: The distance from the flower stalk base to the naturally extended spike apex;

Flower spike diameter: The diameter at the basal segment of the flower stalk, measured 1 cm above the spike base.

For each trait, measurements were replicated across all plants within each biological replicate, and the mean values were calculated for subsequent statistical analysis.

### 4.3. Paraffin Section Preparation

At the S2 stage (initial flowering), floral bud tissues were collected from plants in different treatment groups for morphological and anatomical analysis. High-resolution stereomicroscopic images of floral bud samples were acquired using an HP G4050 scanner at a resolution of 4800 dpi to characterize morphological traits. For anatomical sectioning, floral bud tissues were first fixed in FAA fixative (formalin-acetic acid-ethanol solution, *v*/*v*/*v* ratio as standard), then fixed samples were dehydrated through a graded ethanol series (concentrations specified per standard protocols), post-dehydration samples were transferred to an ethanol-xylene mixture for 10 min with two consecutive treatments for clearing, followed by immersion in molten paraffin and incubation at 65 °C for paraffin infiltration, infiltrated samples were then embedded in pure paraffin and cooled on a −20 °C freezing platform (Model JB-L5, Junjie Electronics, Wuhan, China) to form paraffin blocks, which were vertically sectioned at a thickness of 5 μm using a microtome (Model RM2016, Leica Instruments, Shanghai, China), and finally the sections were stained with toluidine blue dye (Cat. No. G1032, Servicebio, Wuhan, China) and observed and imaged under an optical microscope (Model Eclipse E100 with DS-U3 camera, Nikon Instruments (Shanghai) Co., Ltd., Shanghai, China) [24].

### 4.4. Measurement of Leaf Physiological Indicators

Collected *Phalaenopsis* leaf samples were rinsed with deionized water and stored at −80 °C. For soluble sugar determination: 0.2 g of the sample was accurately weighed and homogenized with 1 mL of phosphate buffer (pH 7.8); the homogenate was incubated in a 95 °C water bath for 10 min, cooled, and centrifuged at 6000 rpm; the supernatant was collected and diluted to 10 mL with distilled water. A 200 μL aliquot of the diluted supernatant was mixed with 200 μL distilled water, 100 μL anthrone-ethanol solution, and 1 mL concentrated sulfuric acid; after thorough mixing, the mixture was re-incubated in a 95 °C water bath for 10 min, cooled to room temperature, and the absorbance values of the blank and sample tubes were measured at 620 nm using a UV spectrophotometer. Soluble sugar content was calculated via the formulas: ΔA = A_sample tube − A_blank tube; soluble sugar content (mg·g^−1^) = [(ΔA + 0.07)/8.55 × V_1_]/(W × V_1_/V_2_) = 1.17 × (ΔA + 0.07)/W (where V_1_ = volume of diluted supernatant, V_2_ = volume of supernatant used for dilution, W = fresh weight of sample, g) [47].

For soluble protein (SP) determination: The Coomassie Brilliant Blue G-250 staining method was adopted. A soluble protein standard curve was prepared following Bradford’s protocol; 0.2 g of the sample was homogenized with 8 mL of phosphate buffer (pH 7.8), and the homogenate was centrifuged at 8000 rpm for 20 min. A 0.5 mL aliquot of the supernatant was mixed with 0.5 mL distilled water and 3 mL Coomassie Brilliant Blue reagent; the mixture was incubated in a 30 °C water bath for 20 min, cooled to room temperature, and its absorbance was measured at 595 nm using a UV spectrophotometer. Soluble protein content was calculated via the formula: Soluble protein content (μg·g^−1^) = (C × VT)/(W × VS × 1000) (where C = protein concentration derived from the standard curve, μg; VT = total volume of extract, mL; VS = volume of extract used for measurement, mL; W = fresh weight of sample, g) [47].

Starch content was determined using the anthrone colorimetric method, with minor modifications based on the protocol described by Subroto et al. [48]. Free fatty acid content was measured via the standard colorimetric method using a commercial kit (Nanjing Jiancheng Bioengineering Institute, Nanjing, China) [48].

### 4.5. Measurement of Endogenous Hormone Content

*Phalaenopsis* leaf samples were collected at two developmental stages (S1: spike emergence; S2: initial flowering), immediately frozen in liquid nitrogen, and stored at −80 °C for endogenous hormone quantification. Sample purification was performed using an Oasis HLB solid-phase extraction (SPE) column (60 mg/3 mL, Waters, USA): the column was pre-activated with 3 mL of methanol and 3 mL of water, then the combined supernatant was loaded; after washing with 3 mL of 5% methanol (*v*/*v*), the target hormones were eluted with 3 mL of methanol containing 0.1% formic acid (*v*/*v*). The eluate was concentrated to dryness under nitrogen flow at 35 °C, then re-dissolved in 200 μL of mobile phase (water:acetonitrile = 95:5, *v*/*v*, containing 0.04% acetic acid) and filtered through a 0.22 μm polyvinylidene fluoride (PVDF) membrane for UPLC-MS/MS analysis [13].

Endogenous hormone contents were determined via ultra-high performance liquid chromatography-tandem mass spectrometry (UPLC-MS/MS) by Meiwei Metabolomics Biotechnology Co., Ltd. (Wuhan, China), with minor modifications to previously established protocols [13]. Targeted endogenous hormones included indole-3-acetic acid (IAA), abscisic acid (ABA), gibberellins (GAs), cytokinins (CKs), jasmonic acid (JA), salicylic acid (SA), and ethylene (ETH) (Supplementary Materials). Hormone identification was achieved by matching the retention time (RT) and characteristic ion pairs of samples with those of standard substances (Sigma-Aldrich, St. Louis, MO, USA) and using the MetWare Database (Meiwei Metabolomics, Wuhan, China), a library specialized in plant endogenous hormone quantification that includes RT, parent ion, and daughter ion information for 150+ plant hormones—for secondary confirmation. For ethylene (ETH), since it is a gaseous hormone, its content was indirectly quantified by detecting its precursor 1-aminocyclopropane-1-carboxylic acid (ACC) using the same extraction and purification protocol, with ACC standard (Sigma-Aldrich, USA) and D_4_-ACC internal standard (10 ng/mL) for calibration.

Chromatographic conditions were set as follows: mobile phase = water-acetonitrile (95:5, *v*/*v*) supplemented with 0.04% acetic acid; flow rate = 0.35 mL·min^−1^; column temperature = 40 °C; injection volume = 2 μL. Hormone detection was performed using a QTRAP 6500 LC-MS/MS system, and raw data were processed with Xcalibur v2.1 software (Thermo Fisher Scientific, Sunnyvale, CA, USA). Each treatment group included three biological replicates to ensure data reliability.

### 4.6. RNA Extraction, Transcriptome Sequencing, and Data Analysis

Total RNA was extracted from orchid leaves subjected to different treatments, and the integrity and purity of the extracted RNA were assessed using the method by Li et al. [25]; each treatment was set with three replicates. The transcriptome sequencing data were completed by Guangzhou Kidiya Biotechnology Co., Ltd. (Guangzhou, China). After sequencing on the Illumina NovaSeq 6000 platform (Illumina, San Diego, CA, USA), low-quality reads were filtered using Trimmomatic, followed by de novo assembly using Trinity software (v2.13.2), resulting in 161,941 unigenes (N50 = 1654 bp). The assembly results were subjected to multi-database joint annotation using DIAMOND BLASTX (e-value ≤ 1 × 10^−5^) and HMMER 3.3.2, with an annotation success rate of 57.00%. Differential expression analysis utilized Salmon for quantifying expression levels, and DESeq2 was employed to identify differentially expressed genes (|log2FC| ≥ 1, FDR < 0.05), followed by functional enrichment analysis using ClusterProfiler. All raw data have been submitted to the NCBI SRA database (accession number: PRJNA1320953).

### 4.7. qRT-PCR Validation

RNA was reverse transcribed into cDNA using the PrimeScript RT reagent kit (RR047A, Tara, Dalian, China), and the cDNA was diluted 10-fold. Gene-specific primers were designed using Oligo7, and published data on Dendrobium loddigesii were selected as reference genes. qRT-PCR was performed using the SYBR Green I Master kit (Roche, Basel, Switzerland), and the relative expression levels of each gene were calculated using the 2^^(−∆Ct)^ method [25].

### 4.8. Statistical Analysis

All data were statistically analyzed using DPS Statistics 7.5, and variance analysis was conducted using Duncan’s new multiple range method. Results are presented as mean ± standard deviation. Statistical significance was set at *p* < 0.05. Graphs and image analyses were conducted using GraphPad Prism 8 software (GraphPad Software, La Jolla, CA, USA) and R software version 3.4.

## 5. Conclusions

**Figure 13:**
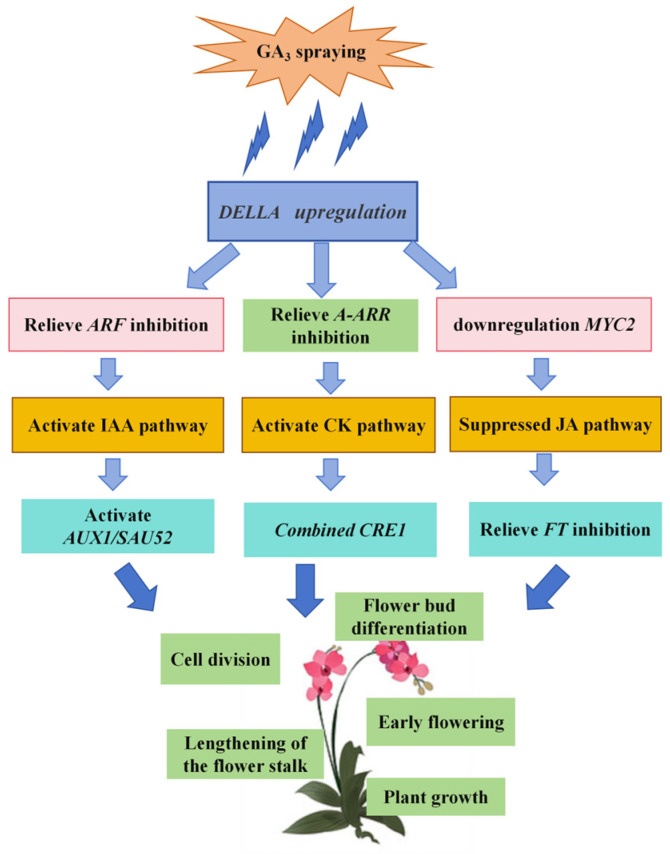
Exogenous GA_3_ spraying regulates the mechanism model of re-flowering in *Phalaenopsis* ‘Hatuyuki’.

In summary, using the commercially valuable *Phalaenopsis* ‘Hatuyuki’ as the experimental plant, we administered exogenous GA_3_ at varying concentrations. Comprehensive analyses of growth phenotypes, physiological responses, endogenous hormonal profiles, and transcriptomic data demonstrated that exogenous GA_3_ application increases endogenous GA levels, synergistically enhances the levels of IAA, CKs, and other endogenous hormones, while decreasing the levels of JA and related hormones. Additionally, this regulatory process activates plant hormonal signaling pathways and involves the differential expression of several genes, including *AUX1*, *IAA*, *SAUR*, *SAUR32*, *DELLA*, *JAZ*, *MYC2*, *PP2C*, *TCH4*, and *TCH4_2*, ultimately promoting reflowering in *Phalaenopsis* ‘Hatuyuki’ (Figure 13). Our findings clarify the core regulatory relationships between exogenous GA_3_, endogenous hormones (GAs, IAA, CKs, JA), and key signaling genes (e.g., *DELLA*, *AUX*, *IAA*, *SAUR*, and *MYC2*) in *Phalaenopsis* ‘Hatuyuki’ reflowering, and inferred critical genes mediating these hormone crosstalk pathways. Exogenous GA_3_ regulates *Phalaenopsis* reflowering via a ‘GA-DELLA-IAA/CK/JA’ crosstalk module, with DELLA serving as a central hub for hormone signal integration. These results not only offer the novel insights of GA-mediated reflowering regulation in *Phalaenopsis* but also offer practical guidance for optimizing industrial cultivation protocols.

## Figures and Tables

**Table 1:** Summary of the transcriptome sequencing data of the 15 libraries constructed using the leaves of *Phalaenopsis* ‘Hatuyuki’ under different GA_3_-treated groups.

Sample	Raw Reads	Clean Reads	Clean Base (G)	Q20 (%)	Q30 (%)	GC Content (%)	Total Mapped Reads (%)	Uniquely Mapped Reads (%)	Multiple Mapped Reads (%)	Known Genes (%)
CK-1	54,841,928	52,869,856	7.93	99.04	96.59	47.32	15.68	12.31	0.12	83.26
CK-2	54,802,054	52,852,302	7.93	99.04	96.61	47.41	19.86	13.65	0.08	77.25
CK-3	52,919,290	50,998,418	7.65	99.11	96.79	47.05	18.87	10.56	0.23	78.56
T1-1	57,579,628	55,103,474	8.27	98.99	96.42	47.07	20.23	15.32	0.06	81.23
T1-2	55,800,648	53,275,878	7.99	99.04	96.56	47.05	18.63	12.33	0.09	74.56
T1-3	62,108,194	59,591,940	8.94	99.14	96.87	47.19	16.48	8.89	0.07	77.52
T2-1	72,932,666	69,883,844	10.48	99.07	96.67	47.03	19.58	10.69	0.06	78.59
T2-2	64,222,592	61,359,334	9.2	99.11	96.77	46.92	20.15	11.25	0.11	80.23
T2-3	71,058,798	68,631,230	10.29	99.22	97.04	47.05	20.36	10.36	0.06	81.06
T3-1	57,826,344	55,470,314	8.32	99.02	96.53	47.66	19.68	10.25	0.09	77.56
T3-2	54,212,068	52,227,802	7.83	99.05	96.65	47.56	18.59	8.56	0.05	81.56
T3-3	63,936,974	61,490,882	9.22	99.06	96.66	47.69	19.65	7.65	0.05	82.21
T4-1	53,961,002	51,805,616	7.77	99.06	96.61	47.03	14.36	4.38	0.08	79.56
T4-2	57,041,832	54,870,944	8.23	99.07	96.65	47.48	13.65	7.66	0.06	78.58
T4-3	65,232,004	62,537,240	9.38	99.15	96.91	46.25	12.24	6.25	0.07	76.55

## Data Availability

The transcriptome sequencing data in this study have all been uploaded to the National Center for Biotechnology Information (NCBI), with access number PRJNA1320953 (https://www.ncbi.nlm.nih.gov/bioproject/PRJNA1320953, accessed on 30 October 2025).

## Supplementary Materials

The following supporting information can be downloaded at: https://www.mdpi.com/article/10.3390/ijms262211069/s1.
